# Aliro: an automated machine learning tool leveraging large language models

**DOI:** 10.1093/bioinformatics/btad606

**Published:** 2023-10-05

**Authors:** Hyunjun Choi, Jay Moran, Nicholas Matsumoto, Miguel E Hernandez, Jason H Moore

**Affiliations:** Department of Computational Biomedicine, Center for Artificial Intelligence Research and Education, Cedars Sinai Medical Center, West Hollywood, CA 90069, United States; Department of Computational Biomedicine, Center for Artificial Intelligence Research and Education, Cedars Sinai Medical Center, West Hollywood, CA 90069, United States; Department of Computational Biomedicine, Center for Artificial Intelligence Research and Education, Cedars Sinai Medical Center, West Hollywood, CA 90069, United States; Department of Computational Biomedicine, Center for Artificial Intelligence Research and Education, Cedars Sinai Medical Center, West Hollywood, CA 90069, United States; Department of Computational Biomedicine, Center for Artificial Intelligence Research and Education, Cedars Sinai Medical Center, West Hollywood, CA 90069, United States

## Abstract

**Motivation:**

Biomedical and healthcare domains generate vast amounts of complex data that can be challenging to analyze using machine learning tools, especially for researchers without computer science training.

**Results:**

Aliro is an open-source software package designed to automate machine learning analysis through a clean web interface. By infusing the power of large language models, the user can interact with their data by seamlessly retrieving and executing code pulled from the large language model, accelerating automated discovery of new insights from data. Aliro includes a pre-trained machine learning recommendation system that can assist the user to automate the selection of machine learning algorithms and its hyperparameters and provides visualization of the evaluated model and data.

**Availability and implementation:**

Aliro is deployed by running its custom Docker containers. Aliro is available as open-source from GitHub at: https://github.com/EpistasisLab/Aliro.

## 1 Introduction

In recent years, the advancement of artificial intelligence (AI) and machine learning (ML) has shown remarkable promise in advancing the field of biomedical and healthcare research, while data in those domains are growing exponentially (Cremin *et al.* 2022). However, the use of these technologies often requires expertise in computer science and programming, which can be a significant barrier for researchers without specialized training. Many data science tasks involve writing code to manipulate, analyze, and visualize data, which can be difficult for beginners or non-programmers.

To address the challenge of ML accessibility to non-experts, we introduce Aliro, a user-friendly and open-source software package that automates ML. Aliro is the successor to an open-source project called PennAI (La Cava *et al.* 2021). Here, we introduce Aliro for AutoML with new tools that leverage the power of large language models (LLMs) (OpenAI 2022) to facilitate and accelerate data analysis by providing a user-friendly web interface that allows users to interact with their data by dynamically executing Python code in the context of a chat or notebook. Users can also customize and modify the code snippets according to their needs and preferences. Aliro also includes a pre-trained machine learning recommendation system that can assist users to automate the selection of machine learning algorithms and their hyperparameters, as well as provide visualization of the evaluated model and data (Olson and Moore 2016).

## 2 Application features

### 2.1 Backend architecture

Aliro is a scalable and modular ML analysis software that leverages Docker containers to provide a lightweight and reproducible environment for its diverse functionalities. By separating its features into three distinct Docker containers—a frontend ReactJS container, a MongoDB database container, and a Python executing container—Aliro achieves a high degree of flexibility and scalability.

The frontend ReactJS container serves as the user interface, enabling interaction with Aliro. It manages tasks such as data input, model training, and visualization by communicating through Application Programming Interfaces (APIs) with the backend containers. Through this communication, Aliro performs model training and executes Python code as a background process.

The database container utilizes MongoDB, a document database, to store datasets, models, and chat conversations. To leverage LLMs, users have the option to provide their own OpenAI API key, establishing a connection to the OpenAI API. The Aliro Controller Engine, a NodeJS server, incorporates a versatile “bridge” API, facilitating communication between the existing Aliro components and the OpenAI API. This bridge API supports all properties outlined in the OpenAI reference guide, enabling Aliro to utilize any available LLMs and endpoints from OpenAI.

The Python executing container is responsible for executing ML analysis tasks and generating code. This container not only facilitates code execution but also incorporates a job scheduling service to manage and prioritize the tasks efficiently. Aliro utilizes a custom-built Docker image that includes the necessary Python libraries for ML analysis and interacts with the language model. This container can be horizontally scaled to handle larger ML analysis tasks or accommodate increased user demand. This container can execute Python scripts generated from conversations between the user and OpenAI. The scripts can be initially provided by OpenAI and subsequently modified by the user. Any required packages can be installed dynamically in the machine backend, granting Aliro the flexibility to handle diverse code. The code execution occurs within the context of the user’s dataset and the trained model, whether manually provided by the user or automatically suggested by Aliro’s recommendation system. This context allows Aliro to answer questions and provide further insights into the existing dataset and experiments.

Aliro’s code execution utility has the capability to save artifacts produced by scripts, such as text and image files, which can be presented as results to the user within the context of the conversation. Aliro does not moderate the code that is executed; code is run as-is and any errors produced by the script are fed back to the web interface. Users then have the option to provide feedback on errors to the LLM for a corrected script or manually edit and rectify the code. While the flexibility in running Python scripts carries potential security risks, Aliro mitigates these concerns by running within Docker containers, which provide an additional layer of security between Aliro and the user’s underlying operating system. All code execution occurs within the container, ensuring no harm is caused to the user’s local machine or operating system. A broken machine component resulting from a malicious script can be easily redeployed by a new container.

The use of Docker containers offers several benefits to Aliro users. Firstly, it ensures the isolation of each application component, minimizing conflicts arising from different dependencies. Secondly, it allows easy horizontal or vertical scaling of each container to meet varying user needs, accommodating high user demand and large datasets effectively. Lastly, Docker containers provide a standardized environment for running ML analysis tasks, enhancing the reproducibility of analyses.

### 2.2 Web interface

**Figure 1.**
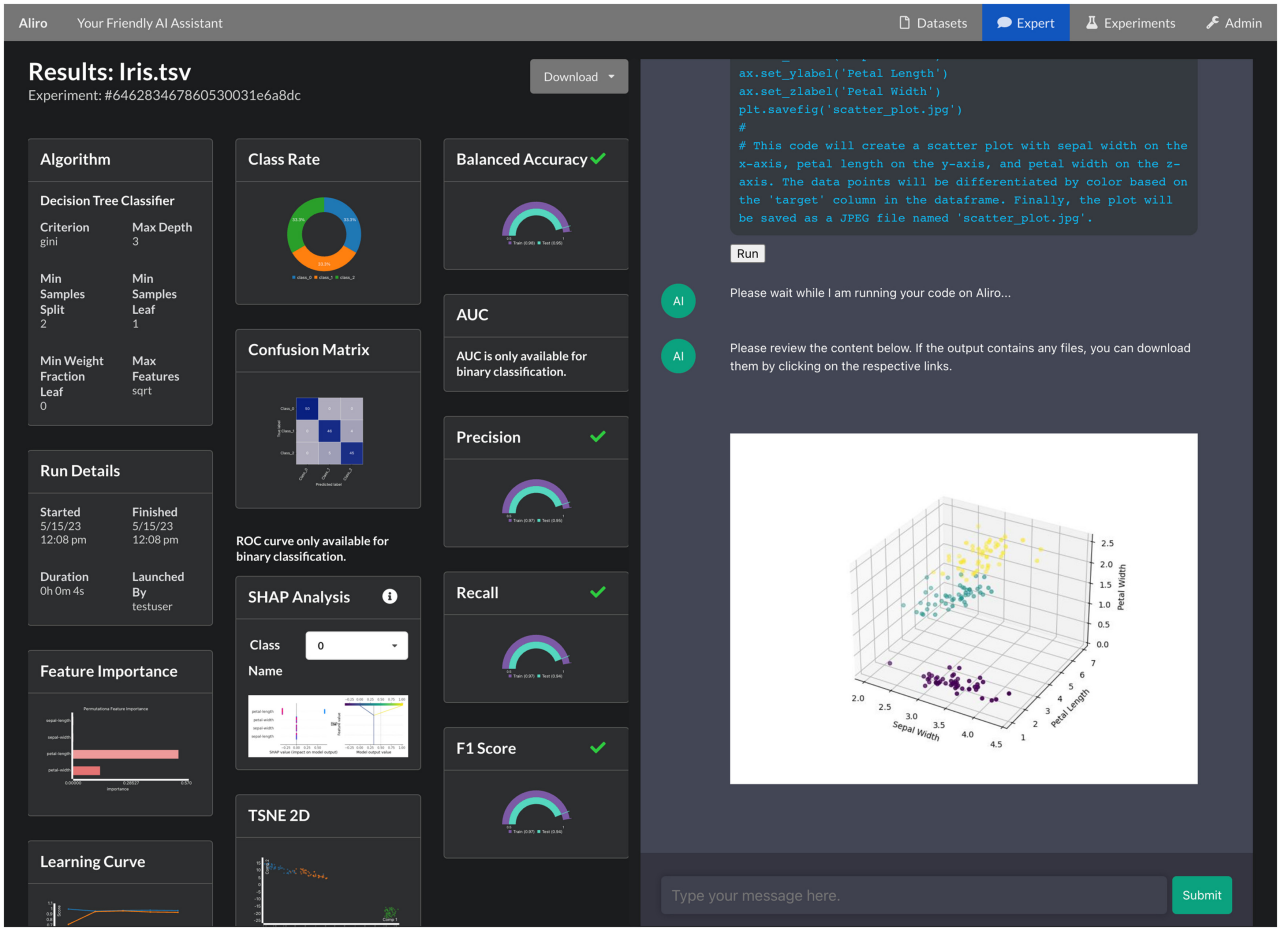
Screenshot of Aliro. The user is provided a split screen of details of the trained model using a variety of visualizations (left) and an LLM-powered, code executable chat where the user requested a 3D scatter plot of the data (right).

Powered by ReactJS, a widely used JavaScript library for building web interfaces, Aliro offers a responsive and interactive web application that enables seamless interaction with its features (see Figure 1).

The workflow of Aliro is intuitive, allowing users of any level to easily navigate through its simple user interface. Starting with dataset uploading, users can select ML algorithms to train models and explore data and model performance through visualizations.

A key aspect of Aliro is its chat-based interface, enabling users to engage with the tool without requiring extensive familiarity with data science concepts. Through API calls to the Python backend and LLM endpoints, this interactive interface provides a conversational experience, allowing users to ask questions and receive relevant insights and guidance, eliminating the need for manual coding and reducing entry barriers to data science tasks.

Another valuable feature is the code editing capability, enabling users to modify and customize the generated code snippets according to their specific needs. This flexibility allows users to tailor the code to suit their analysis requirements, providing a sense of ownership and control over the data science process.

Aliro further facilitates code execution, enabling users to run their code snippets directly within the tool. This integrated functionality saves users from switching between different environments, ensuring a seamless experience. Additionally, Aliro includes features such as package installation, data visualization, and data processing. Users can install required packages directly through the chat interface, ensuring they have the necessary libraries for their data analysis tasks. The visualization feature offers visual representations to help users gain a better understanding of their datasets. Moreover, users can process datasets within Aliro and generate new experiments based on modified or augmented data, enabling iterative analysis and experimentation.

To enhance user experience and facilitate development, Aliro incorporates a submit error button. Users can report any issues or errors they encounter during their interactions with the tool, allowing them to receive fixed code and guidance from the language model. Additional details are provided in the Supplementary Data.

### 2.3 ML recommender system

One of the key features of Aliro is its built-in ML recommender system. This system aims to assist users in automating the selection of ML algorithms and their hyperparameters, streamlining the model selection process and accelerating the discovery of insights from their data. The ML recommender system selects personalized recommendations based on the characteristics of the user’s dataset and their specific objectives. By analyzing the dataset’s properties, such as the number of features, the distribution of data, and the target variable, the recommender system can suggest suitable ML algorithms that are likely to perform well on the given data.

This feature is especially useful for those unfamiliar with machine learning algorithms and the nuances behind the hyperparameters for any given algorithm. With a simple toggle, the user will be able to train multiple recommended models concurrently and subsequently select the best model from the list of evaluated models for their dataset.

## 3 Installation

To install Aliro, the only requirement is to have Docker installed on the host machine. Docker is a widely used containerization platform that allows applications to run in isolated environments, ensuring consistent behavior across different systems. Users can simply build the custom Aliro images, and run it using Docker commands. The Aliro Docker images contain all the necessary components, including the frontend React container, the database container, and the Python execution container. Further documentation about installation and usage is found in the Aliro Github: Aliro User Guide.

## 4 Example use cases

To illustrate the practical application of Aliro, we present a use case involving an Alzheimer’s disease dataset. In this example, researchers aim to analyze the dataset, build a model, improve data quality, and explore the potential for data enrichment (Urbanowicz *et al.* 2017).

Researchers begin by uploading the Alzheimer’s disease dataset into Aliro’s datasets page. Once the dataset is uploaded, they proceed to build their first model using a machine learning algorithm of their choice, such as a random forest classifier. After building the model, researchers navigate to Aliro’s Experiment page to gain insights into model performance and dataset characteristics. Aliro presents visually intuitive metrics, including accuracy, precision, recall, and a confusion matrix, enabling researchers to evaluate the model’s effectiveness. Furthermore, Aliro generates feature importance values, such as SHAPley values (Lundberg and Lee 2017), to identify potential influential features in predicting Alzheimer’s disease. Additionally, researchers can visualize the dataset using t-distributed Stochastic Neighbor Embedding (tSNE) and Principal Component Analysis (PCA) scatterplots, which are unsupervised methods of determining patterns within the dataset.

Say while exploring the PCA scatterplot, researchers notice the presence of outliers that might affect model performance. To address this, they can engage in a conversation with Aliro’s chat feature within their Experiment page. Researchers inquire about outlier detection methods and request code snippets to identify and remove outliers. The LLM will generate the code, which can be executed with a single click using Aliro’s run button for that code block. With the new modified dataset without outliers, the researcher can upload this as a new dataset, and train new models.

In the pursuit of enhancing the Alzheimer’s disease dataset, researchers can connect Aliro to a knowledge graph, AlzKB, a public Neo4j database specializing in Alzheimer’s disease and via the chat feature, instruct Aliro to pull relevant data from the AlzKB database and integrate it with their own dataset. This enrichment step provides additional information that can provide more signal for analysis of Alzheimer’s disease. By iteratively analyzing the data and refining the models, researchers can uncover new insights and potentially improve the accuracy of predictions and determine which enrichment strategies perform best.

In this example, Aliro showcases its ability to streamline the data analysis workflow for Alzheimer’s disease research. By utilizing its various features, including model evaluation, outlier detection and removal, data visualization, enrichment through knowledge graphs, and iterative analysis, Aliro empowers researchers to extract meaningful information from complex datasets, enhance data quality, and make advancements in understanding their dataset.

## 5 Conclusion

Aliro represents a significant step forward in the development of user-friendly, open-source AI tools for data science research. With Aliro, users can easily upload datasets, select models to train, and explore their data through intuitive visualizations. By offering a conversational experience, Aliro allows users to ask questions, seek guidance, and receive relevant insights through a chat. The novel interactive experience of Aliro lies in its ability to generate and run code specific to the user’s data and model, leveraging the power of the LLMs to provide a tailored and dynamic code execution environment. The ML recommender system further simplifies the process by automating the selection of ML algorithms and their hyperparameters, eliminating the need for users to have in-depth knowledge of different algorithms. This empowers users without a background in machine learning to effectively perform data analysis tasks and make informed decisions based on the recommendations provided by Aliro. By reducing the barriers to entry and providing a seamless user experience, Aliro opens up opportunities for individuals from various domains to explore and leverage the power of machine learning in their work or research.

## Supplementary Material

btad606_Supplementary_Data
