# Supplementary material for: Aliro: an automated machine learning tool leveraging large language models
Source: Bioinformatics. 2023 Oct 5;39(10):btad606. doi: 10.1093/bioinformatics/btad606 (PMC10576163; doi:10.1093/bioinformatics/btad606)
Supplement: btad606_Supplementary_Data [file btad606_supplementary_data.zip › Aliro_Application_Note_Supplementary (2).pdf]

Supplementary Material for Aliro

Hyunjun Choi, Jay Moran, Nicholas Matsumoto,
Miguel E. Hernandez, and Jason H. Moore

September 22, 2023

1 Introduction

The provided document is the supplementary material related to the article titled *Aliro: an Automated Machine Learning Tool Leveraging Large Language Models*. This supplementary material discusses additional documentation and clarification that is described in the main article. For complete documentation, the user can go to: https://epistasislab.github.io/Aliro/userguide.html

Section 2 contains information about uploading a dataset and what is required for the dataset for it to be processed properly by Aliro. Section 3 contains information about removal of datasets.

2 Uploading a dataset

Aliro 1.0 has specific dataset requirements since it is designed to focus on downstream machine learning and interactive visualization rather than data preprocessing tasks like imputation or one-hot encoding. These requirements are:

- Datasets must have the extension .csv or .tsv
- Datasets cannot have any null or empty values
- Dataset values must be numeric. All categorical features must be encoded to a numeric value prior to upload.
- Files must be smaller than 8MB

There are public datasets available within the GitHub repository of Aliro that satisfy the above requirements:

https://github.com/EpistasisLab/Aliro/tree/master/data/datasets/pmlb_small

Additionally, in the supplementary material, we have supplied a sample "iris" classification dataset that is Aliro-compatible as well as an "iris_outlier" dataset

that includes outliers in the dataset that the user can remove through the Chat feature an an exercise.

The dataset should also have a sufficient sample size due to algorithmic restrictions in certain machine learning methods. For example, the *neighbors* hyperparameter for K-Nearest Neighbors classifiers requires the sample size to be larger than the number of neighbors. As a rule of thumb, having more than 10 samples for the dataset should be suitable for the machine learning algorithms provided by Aliro 1.0.

3 Deleting datasets

In version 0.20, removing datasets in Aliro requires the user to execute a Docker command that essentially reboots the cache and database systems. You can achieve this by running the following command: "docker-compose up –force-recreate." This command effectively refreshes the MongoDB database container, where all the essential dataset information is stored. For additional information and step-by-step guidance, please consult our online user guide. It's important to note that the ability to remove individual datasets will be introduced as a forthcoming feature in version 0.21.
